# Prediction of Anticancer Peptides Using a Low-Dimensional Feature Model

**DOI:** 10.3389/fbioe.2020.00892

**Published:** 2020-08-12

**Authors:** Qingwen Li, Wenyang Zhou, Donghua Wang, Sui Wang, Qingyuan Li

**Affiliations:** ^1^College of Animal Science and Technology, Northeast Agricultural University, Harbin, China; ^2^Center for Bioinformatics, School of Life Sciences and Technology, Harbin Institute of Technology, Harbin, China; ^3^Department of General Surgery, Heilongjiang Province Land Reclamation Headquarters General Hospital, Harbin, China; ^4^Key Laboratory of Soybean Biology in Chinese Ministry of Education, Northeast Agricultural University, Harbin, China; ^5^State Key Laboratory of Tree Genetics and Breeding, Northeast Forestry University, Harbin, China; ^6^Forestry and Fruit Tree Research Institute, Wuhan Academy of Agricultural Sciences, Wuhan, China

**Keywords:** anticancer peptide, feature extraction, feature model, feature selection, machine learning

## Abstract

Cancer is still a severe health problem globally. The therapy of cancer traditionally involves the use of radiotherapy or anticancer drugs to kill cancer cells, but these methods are quite expensive and have side effects, which will cause great harm to patients. With the find of anticancer peptides (ACPs), significant progress has been achieved in the therapy of tumors. Therefore, it is invaluable to accurately identify anticancer peptides. Although biochemical experiments can solve this work, this method is expensive and time-consuming. To promote the application of anticancer peptides in cancer therapy, machine learning can be used to recognize anticancer peptides by extracting the feature vectors of anticancer peptides. Nevertheless, poor performance usually be found in training the machine learning model to utilizing high-dimensional features in practice. In order to solve the above job, this paper put forward a 19-dimensional feature model based on anticancer peptide sequences, which has lower dimensionality and better performance than some existing methods. In addition, this paper also separated a model with a low number of dimensions and acceptable performance. The few features identified in this study may represent the important features of anticancer peptides.

## Introduction

Cancer is still a severe health problem globally, and lots of people have died from cancer (Liao et al., 2018; Cheng et al., 2019a; Zeng W. et al., 2019; Zhang Y. et al., 2019; Zhou et al., 2019; Yang et al., 2020). Traditional cancer treatments kill not only cancer cells but also normal cells, and the medical costs are very high (Feng, 2019; Lin et al., 2019; Li Y.H. et al., 2020; Zhang et al., 2020). With the find of anticancer peptides, the situation has changed because anticancer peptides can interact with the anionic cellular elements of cancer cells to selectively kill cancer cells without harming the normal cells of the body (Ozkan et al., 2019; Wang Y. et al., 2020; Yin et al., 2020). Although there have been some defects in the development of anticancer peptides, anticancer peptides are safer than man-made drugs (Sun et al., 2016; Liu H. et al., 2018; Liao and Jiang, 2019; Munir et al., 2019; Srivastava et al., 2019; Liu H. et al., 2020; Ru et al., 2020; Wang J. et al., 2020) and have higher effectiveness, specificity and selectivity. Anticancer peptides provide a new direction for the treatment of cancer, so the therapeutic methods of anticancer peptides have attracted greater attention. Anticancer peptides are generally composed of five to thirty amino acids. Nevertheless, it is still hard to identify anticancer peptides from other (artificially designed or natural) peptides. Using biochemical experiments to identify anticancer peptides is very time-consuming and expensive. In addition, only a few anticancer peptides can be used in the clinic. Thus, it is essential to apply machine learning to forecast anticancer peptides.

In past few years, some bioinformatics methods have been introduced to predict anticancer peptides. By extracting the amino acid composition and binary features of anticancer peptides as feature vectors, Tyagi et al. (2013) applied support vector machine to verify the performance, and the accuracy reached 91.44%. Hajisharifi et al. (2014) applied support vector machine to predict anticancer peptides on the basis of the local alignment kernel and pseudo-amino acid composition, and the highest accuracy was 89.7%. Chen W. et al. (2016) developed a classifier for predicting anticancer peptides by optimizing the composition of g-GAP dipeptides, and 94.77% accuracy was obtained by using 126D features. Xu et al. (2018b) used 400D features or 400D-g gap features to predict anticancer peptides, and the accuracy of support vector machine reached 91.86%. The above methods obtained sound prediction results, but these methods did not mention the dimensional advantages of the model. In reality, training the machine learning model utilizing high-dimensional features usually behaves poorly, This phenomenon is called Curse of Dimensionality (Wilcox, 1961; Xu et al., 2017; Xu Y. et al., 2018; Zou et al., 2017; Wang et al., 2019).

In this paper, through using a variety of polypeptide feature extraction methods, the obtained feature vectors were selected many times, which gained a low-dimensional model. Using multiple classifiers for verification, the performance accuracy was 92.73%, while the number of dimensions of the model was only 19. In this paper, the most important 7 dimensional features were further separated and verified, and good results were obtained. The feature model obtained in this paper can not only accurately and rapidly classify anticancer peptides, but also effectively avoid Curse of Dimensionality. The above results may suggest that these low-dimensional features are important features for distinguishing anticancer peptides.

## Materials and Methods

**FIGURE 1:**
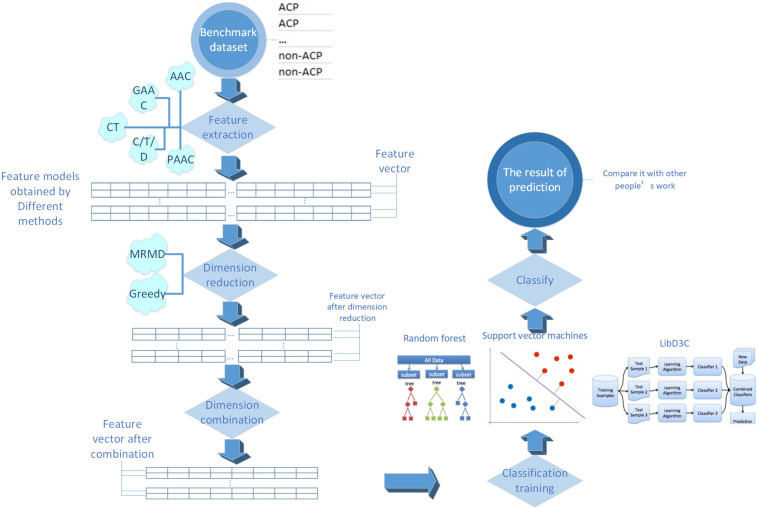
The main flow chart of the research process in this paper.

The process of this research is shown in Figure 1. Every detailed step will be presented in the following sections.

### Benchmark Dataset

In this paper, we used the benchmark dataset constructed by Hajisharifi et al., which contained 206 non-anticancer peptides and 138 anticancer peptides. The anticancer peptides in this data set were extracted from APD2, and 206 non-anticancer peptides established by Wang et al. were extracted from UniProt. To avoid the deviation of the classifier, peptides with more than 90% similarity were deleted from the data set through CD-HIT. Chen et al. and Xu et al. have applied the identical benchmark data set as well.

### Feature Extraction Strategies

The peptide sequences can not be immediately identified by machine learning algorithms. Therefore, it is requisite to translate the strings stood for peptide sequences into numerical features (Liu et al., 2006, 2019b; Liu S. et al., 2018; Jia et al., 2018; Wang et al., 2018; Chen C. et al., 2019; Hong J. et al., 2019). The feature extraction methods are very crucial in building computational predictors (Cheng et al., 2018, 2019b; Xiong et al., 2018; Zhang et al., 2018b,2019a; Sun et al., 2019; Tang et al., 2019).

In this paper, we applied five sorts of feature extraction strategies including amino acid composition (AAC), conjoint triad (CT), pseudo-amino acid composition (PAAC), grouped amino acid composition (GAAC) and C/T/D. Each strategy may also include several feature extraction methods. This paper implemented these strategies through iFeature (Chen et al., 2018).

#### Conjoint Triad

Shen et al. (2007) put forward the conjoint triad model (CT). In consideration of the properties of one amino acid and its nearby amino acids and regards any three sequential amino acids as a unit, the model classifies amino acids into seven sorts. Triad in the same class are considered similar. As an example, triads which are composed by three amino acids belonging to the same sort, such as GLM and VFT, could be treated equally, since they may play the same role. A peptide sequence is represented by a binary space (V,F). V is the vector space of sequence features. Each feature (v_*i*_) represents a unit. F is the frequency vector corresponding to V, and each feature (f_*i*_) is the frequency of v_*i*_ in a peptide sequence.

#### C/T/D

Dubchak et al. (1995) put forward the C/T/D model. This model considers 3 properties of amino acids, their solubility, secondary structure and relative hydrophobicity. Amino acids are classified into three classes on the basis of the relative hydrophobicity, three or four classes on the basis of the secondary structure, and two classes on the basis of solubility. Each class is presented by the three kinds of descriptors: C/T/D (Tan et al., 2019).

#### Amino Acid Composition

The peptide is composed of 20 sorts of amino acids (Liu et al., 2019a). The frequency of every amino acid type in a peptide sequence was computed to present the peptide sequences. Therefore, each peptide sequence can be represented as a 20-dimensional feature model. This model is called amino acid composition model (AAC). The features can be defined as:

f(a)=NaN, a∈(A,C,...,W,Y)

where N_*a*_ is the quantity of amino acid type a. while N is the length of a peptide sequence.

In this paper, we also used the k-spaced amino acid pair composition model (CKSAAP), which computes the frequency of amino acid pairs separated by an arbitrary number (k) of amino acid residues. A example of this encoding scheme (*k* = 0) is provided as follow:

a⁢peptide⁢sequence:CRACRKDSMVN

The features (*k* = 0) can be defined as:

(NAA=0(N-1),NAC=1(N-1),...,NCQ=0(N-1),NCR=2(N-1),...,NYY=0(N-1))400

At the same time, this paper used the tripeptide composition model (TPC), which computes the frequency of three consecutive amino acids in a peptide sequence and provides 8000 dimensional features. The features can be defined as:

f(a,b,c)=Nabc(N-2),  a,b,c∈(A,C,...,W,Y)

where N_*abc*_ is the quantity of amino acid type a, b, and c. while N is the length of a peptide sequence.

At the same time, this paper used the dipeptide composition model (DPC), which computes the frequency of two consecutive amino acids in a peptide sequence and provides 400D features. The features can be defined as:

f(a,b)=Nab(N-1),  a,b∈(A,C,...,W,Y)

where N_*ab*_ is the quantity of amino acid type a and b. while N is the length of a peptide sequence.

### Pseudo-Amino Acid Composition

Chou (2001) put forward a pseudo-amino acid composition model (PAAC). In this model, It takes into account not only the frequency of each amino acid type in a peptide sequence but also the position information of the amino acids. Therefore, the feature of the pseudo-amino acid composition is stated as below:

PAAC = (a_1_,a_2_,…,a_19_,a_20_,a_20+1_, a_20+2_,…,a_20+*n*_)

The front portion a_1_,…, a_19_,a_20_ stand for the frequency of each amino acid type in a peptide sequence, and the latter portion a_20+1_,…,a_20+*n*_ represent the location info of the amino acids in a peptide sequence.

This paper also used a method similar to PAAC. The amphiphilic pseudo-amino acid composition model (APAAC) was put forward by Chou et al. The model takes the hydrophilic and hydrophobic properties of amino acids into account.

#### Grouped Amino Acid Composition

The grouped amino acid composition model (GAAC) divides 20 amino acid types into 5 classes on the basis of the physical and chemical properties and then computes the frequency of each amino acid group in a peptide sequence. The features can be defined as:

f(c)=NcN,  c∈(c1,c2,c3,c4,c5)

where N_*c*_ is the quantity of amino acid in class c. while N is the length of a peptide sequence.

In this paper, a model similar to the grouped amino acid model, k-spaced amino acid group pair (CKSAAGP), was used to compute the frequency of amino acid group pairs separated by an arbitrary number (k) of amino acid residues.

This paper also used the grouped dipeptide composition model (GDPC), which can be regarded as a combination of GAAC and DPC.

In addition, this paper used the grouped tripeptide composition model (GTPC), which can be regarded as a combination of GAAC and TPC.

### Feature Selection

Feature selection is the procedure of picking out a subset from the relevant features applied in machine learning model building (Zou et al., 2016; Qiao et al., 2018; Cheng, 2019; Yang et al., 2019; Zhang M. et al., 2019; Li F. et al., 2020). The dimension of features will be decreased after feature selection, thus this procedure is named dimension reduction as well. MRMD2.0 was mainly used in this paper to reduce the feature dimensions. Each feature was given a numerical value by MRMD2.0 (the larger the number, the feature’s recognition ability will be more obvious). MRMD2.0 sorted the features in order on the basis of the ranking value. Next, the first feature with the highest value was examined for its performance. The second feature was added to examine the capability of the new feature subset. This procedure continued till examining total features. Eventually, some parameters in disparate dimensions were acquired, including F-score, accuracy, etc.

### Classifier

#### Support Vector Machine

A support vector machine (SVM) was used for prediction in this study. SVM has been widely applied in the proteome prediction (Jiang et al., 2013; Wei et al., 2016, 2018; Ding et al., 2017; Lin et al., 2017; Qu et al., 2017; Wang et al., 2017, 2018; Guo and Xu, 2018; Xu et al., 2018a, b; Zhang et al., 2018a; Chao et al., 2019; Chen Z. et al., 2019; Fang et al., 2019; Hong Z. et al., 2019; Liu and Li, 2019; Yu and Gao, 2019; Zeng et al., 2019b; Dao et al., 2020; Huang et al., 2020), transcriptome (Chen X. et al., 2016; Tang et al., 2017) and genome (Zeng et al., 2017; Song et al., 2018; Deng et al., 2019b; Hong Z. et al., 2019). Therefore, support vector machine is a pretty useful classifier. libSVM was adopted in this paper to optimize the prediction results of SVM utilizing grid method to correct parameters g and c.

#### Random Forest

Random forest (rf) has been extensively applied as a classifier in chemoinformatics (Zeng et al., 2019b,2020a,b; Song et al., 2020) and bioinformatics (Zhang J. et al., 2016; Guo and Xu, 2018; Deng et al., 2019a; Liu et al., 2019a; Lv H. et al., 2019; Lv Z. et al., 2019; Lv et al., 2020; Ru et al., 2019; Wei et al., 2019; Xu et al., 2019; Tang et al., 2020; Yu et al., 2020). Rf was applied in this paper.

#### LibD3C

At the same time, this paper used the LibD3C classifier (Lin et al., 2014) for prediction to examine the performance of the model. The classifier adopts the strategy of selective integration, based on the hybrid integrated pruning model on the basis of k-means clustering and functional selection cycle framework and sequential search, by training multiple classifiers and selecting a group of accurate and diversified classifiers to solve the problem.

### Prediction Result Estimate

It is extremely critical to quantitatively evaluate the effectiveness of the method because the benchmark data set is non-balanced data. This paper used Mathew correlation coefficient (Mcc), specificity (Sp),sensitivity (Sn), total accuracy (Acc) and the F-score value (F-score) phase to evaluate the performance of the model (Li et al., 2015, 2017; Wei et al., 2017; Chu et al., 2019; Ding et al., 2019; Gong et al., 2019; Liang et al., 2019; Shan et al., 2019; Yan et al., 2019; Yu and Gao, 2019; Zeng et al., 2019a,2020b; Zhang et al., 2019b; Liu X. et al., 2020; Wang H. et al., 2020).

Mcc=(TP×TN-FP×FN)/

$$\sqrt{\left(\text{TP}+\text{FP}\right)\times \left(\text{TP}+\text{FN}\right)\times \left(\text{TN}+\text{FP}\right)\times \left(\text{TN}+\text{FN}\right)}$$

Sn=TP/(TP+FN)

Sp=TN/(TN+FP)

Acc=(TP+TN)/(TP+TN+FP+FN)

F-score=2×P×R/(P+R)

where TP stands for the quantity of anticancer peptides correctly predicted, FP stands for the quantity of non-anticancer peptides predicted as anticancer peptides, TN stands for the correctly predicted quantity of non-anticancer peptides, and FN stands for the quantity of anticancer peptides predicted as non-anticancer peptides. P represents the accuracy, indicating the proportion of the total number of predicted positive cases; R is the recall rate, indicating the number of correct cases identified and accounting for the total number of cases in this category.

## Results and Discussion

**FIGURE 2:**
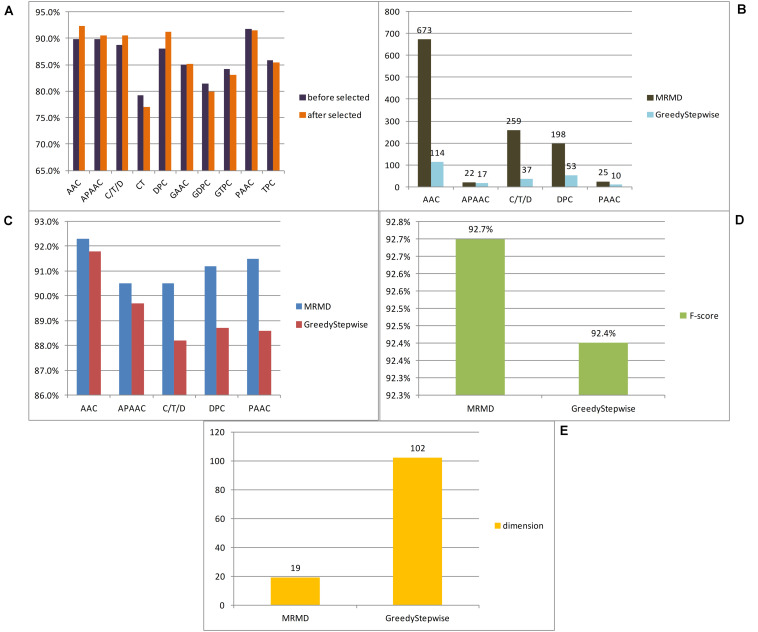
The results of different experiments. **(A)** According to the results, this paper thought that the CT, GAAC, GDPC, GTPC, and TPC are not ideal. **(B)** According to the results, this paper thought that the greedy algorithm was more efficient than MRMD2.0. **(C)** According to the results, this paper thought that the greedy algorithm is worse than MRMD2.0 in the performance index of the selected model. **(D)** After several dimension reductions, the results showed that the MRMD2.0 was better than the greedy algorithm index of the selected model. **(E)** After several dimension reductions, the results showed that the dimension of model of the greedy algorithm is about five times that of the MRMD2.0. The results showed that as for the dimensions of the selected model, the greedy algorithm was more efficient than MRMD2.0. However, the greedy algorithm cannot further reduce the dimensions of the selected feature model, but MRMD2.0 can still further reduce it.

In this paper, a total of 12 feature extraction methods were used. Because the number of dimensions of the amino acid composition model was only 20, it is of little significance to reduce the dimensionality of the amino acid composition model alone, and the k-spaced amino acid pair composition model is an extension of this method. The principles of the two models were similar, and so the two models were merged and expressed uniformly by AAC. Similarly, the grouped amino acid composition model and the k-spaced amino acid group pair model were merged and expressed uniformly by GAAC. To compare the advantages and disadvantages of different feature extraction methods for anticancer peptide sequences, each model obtained by each method was examined by 10-fold cross-validation utilizing the random forest classifier, and then 10-fold cross-validation was carried out for each method after dimensional reduction through MRMD2.0. Figure 2A lists the F-score of each feature extraction method before and after feature selection. In this paper, according to the verification results, it is believed that the effects of the CT, GAAC, GDC, GTC, and TC methods were not ideal, so the above model was not considered in the follow-up study. To compare the advantages and disadvantages of different feature selection methods, the greedy algorithm and MRMD2.0 were used to select each feature model. Figure 2B lists the dimensions of each feature model after two kinds of software selection, and Figure 2C lists the F-score of each feature model after two kinds of software selection. For the feature selection method of anticancer peptide, after synthesizing the situation of all types of model selection, MRMD2.0 was better than the greedy algorithm in terms of the capability index of the selected model; As for the dimensions of the selected model, the greedy algorithm was more efficient than MRMD2.0. However, the greedy algorithm cannot further reduce the dimensions of the selected feature model, but MRMD2.0 can still further reduce it.

The feature subset of each method was merged and reduced to get a 102D feature model after selected by the greedy algorithm. The F-score value was 0.924 after random forest 10-fold cross-validation. At this time, it was impossible to use the greedy algorithm to further reduce the dimensions of the model.

After merging the selected feature model by MRMD2.0, the model dimension number was 1177. This paper continued to use MRMD2.0 to reduce the dimension of the model to get a 767-dimensional feature model which was still too high. After continuing to reduce the dimensionality of the model again to obtain 633 dimensional features, the result was still not ideal. In this paper, the dimensionality reduction was carried out 6 times. For each dimensionality reduction, a line chart of F-score was drawn changing with the dimension according to the obtained indicators. The feature points were separated with large changes in the line to form a new model for verification, and the results were not ideal. After 8 times of dimensionality reduction, a 19-dimensional feature model was obtained. At this time, it was no longer possible to use MRMD2.0 for dimensionality reduction. Figures 2D,E list the feature model F-score and dimensions separated by the two methods, respectively. By comparison, MRMD2.0 was found to be better than the greedy algorithm.

The 19-dimensional model was tested by random forest, support vector machine (parameters c and g are 8192.0 and 0.00048828125, respectively) and LibD3C, respectively. Table 1 listed the prediction results of three types of classifiers. The results indicated that the performance of the 19-dimensional model separated in this paper is stable. Table 1 also lists the prediction results of others based on the same data set. Compared with Hajisharifi et al.’s and Xu et al.’s models, the model in this paper performs better in all prediction indicators. Although it is slightly inferior to Chen et al. in the prediction results, the number of dimensions of their model was 126, while the number of dimensions of this paper is 19, which is obviously lower than that in the previous study. By evaluating the performance of the model and comparing it with the previous work, this paper believed that the 19-dimensional model proposed in this paper can be used to predict the anticancer peptide conveniently, quickly and accurately.

**TABLE 1:** Comparing the performance of different methods.

Methods	Sn	Sp	Acc	MCC	F-score	Dimension
iACP	88.40%	99.02%	94.77%	89.30%		126
Hajisharifi et al.	85.18%	92.68%	89.70%	78.40%		
SAP	86.23%	95.63%	91.86%	83.01%	89.47%	400
Our method(RF)	86.20%	97.10%	92.73%	84.90%	92.70%	19
Our method(LibD3C)	85.50%	96.60%	92.15%	83.70%	92.10%	19
Our method(SVM)	87.70%	96.10%	92.73%	84.80%	92.70%	19

**FIGURE 3:**
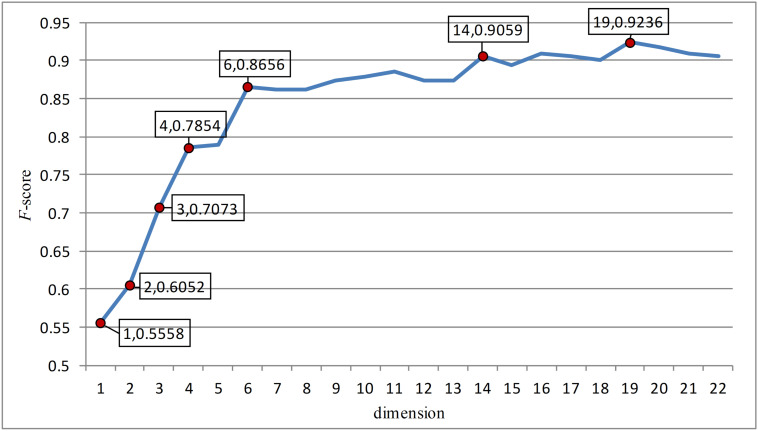
The figure was the change of F-score with dimension according to the last dimension reduction. The red dots in the figure were the feature points with great changes in this paper. And these points were separated to form a new feature model and verified. After verification, these seven red dots are the most important seven features.

In this paper, the feature points with large slopes in the last reduced-dimension line chart (Figure 3) were separated to form a 7-dimensional model, which was verified by support vector machine with an accuracy of 90.41%. This possibly imply that these seven-dimensional features are important features to distinguish anticancer peptides. These 7-dimensional features are GL.gap4, hydrophobicity_PRAM900101.Tr2332, polarizability.2.residue0, Pc1.C, Xc1.K, Pc2.Hydrophobicity.8, and secondarystruct.1.residue0. These features may suggest that for anticancer peptides, the composition and content of glycine, leucine, cysteine and lysine as well as their secondary structure, polarization and hydrophobicity are important indicators different from other non-anticancer peptides.

## Conclusion

In this paper, a low-dimensional feature model with better performance was obtained through feature extraction and continuous feature selection over many iterations. The features were further isolated, and a few features that might distinguish anticancer peptides were identified. It is hoped that the results of this paper can be used in the artificial design and prediction of anticancer peptides.

## Data Availability Statement

The raw data supporting the conclusions of this article will be made available by the authors, without undue reservation.

## Author Contributions

QWL and SW conceived and designed the research. QWL and WZ performed the machine learning experiments. QWL, DW, and QYL analyzed the data. QWL and WZ wrote the manuscript. QYL and SW coordinated the study and revised the manuscript. All authors read and approved the final manuscript.

## Conflict of Interest

The authors declare that the research was conducted in the absence of any commercial or financial relationships that could be construed as a potential conflict of interest.

## References

[B1] Chao L. Wei L. Zou Q. (2019). SecProMTB: a SVM-based classifier for secretory proteins of mycobacterium tuberculosis with imbalanced data set. *Proteomics* 19:e1900007. 10.1002/pmic.201900007 31348610

[B2] Chen C. Zhang Q. Ma Q. Yu B. (2019). LightGBM-PPI: predicting protein-protein interactions through LightGBM with multi-information fusion. *Chemometr. Intellig. Lab. Syst.* 191 54–64. 10.1016/j.chemolab.2019.06.003

[B3] Chen Z. Zhao P. Li F. Marquez-Lago T. Leier A. Revote J. (2019). iLearn: an integrated platform and meta-learner for feature engineering, machine learning analysis and modeling of DNA, RNA and protein sequence data. *Briefings Bioinform.* 21 1047–1057. 10.1093/bib/bbz041 31067315

[B4] Chen W. Ding H. Feng P. Lin H. Chou K.-C. (2016). iACP: a sequence-based tool for identifying anticancer peptides. *Oncotarget* 7:7815. 10.18632/oncotarget.7815 26942877 PMC4941358

[B5] Chen X. Pérez-Jiménez M. J. Valencia-Cabrera L. Wang B. Zeng X. (2016). Computing with viruses. *Theoret. Computer Sci.* 623 146–159.

[B6] Chen Z. Zhao P. Li F. Leier A. Marquez-Lago T. T. Wang Y. (2018). iFeature: a Python package and web server for features extraction and selection from protein and peptide sequences. *Bioinform. J.* 34 2499–2502. 10.1093/bioinformatics/bty140 PMC6658705 29528364

[B7] Cheng L. (2019). Computational and biological methods for gene therapy. *Curr. Gene Ther.* 19 210–210. 31762421 10.2174/156652321904191022113307

[B8] Cheng L. Jiang Y. Ju H. Sun J. Peng J. Zhou M. (2018). InfAcrOnt: calculating cross-ontology term similarities using information flow by a random walk. *BMC Genomics* 19(Suppl. 1):919. 10.1186/s12864-017-4338-6 29363423 PMC5780854

[B9] Cheng L. Yang H. Zhao H. Pei X. Shi H. Sun J. (2019a). MetSigDis: a manually curated resource for the metabolic signatures of diseases. *Brief Bioinform.* 20 203–209. 10.1093/bib/bbx103 28968812

[B10] Cheng L. Zhao H. Wang P. Zhou W. Luo M. Li T. (2019b). Computational methods for identifying similar diseases. *Mol. Ther. Nucleic Acids* 18 590–604. 31678735 10.1016/j.omtn.2019.09.019 PMC6838934

[B11] Chou K.-C. (2001). Prediction of protein cellular attributes using pseudo-amino acid composition. *Proteins* 43 246–255. 10.1002/prot.1035 11288174

[B12] Chu Y. Kaushik A. C. Wang X. Wang W. Zhang Y. Shan X. (2019). DTI-CDF: a cascade deep forest model towards the prediction of drug-target interactions based on hybrid features. *Brief Bioinform.* 2019:bbz152. 10.1093/bib/bbz152 31885041

[B13] Dao F. Y. Lv H. Zulfiqar H. Yang H. Su W. Gao H. (2020). A computational platform to identify origins of replication sites in eukaryotes. *Brief Bioinform*. 2020:bbaa017. 10.1093/bib/bbaa017 32065211

[B14] Deng L. Li W. Zhang J. (2019a). “LDAH2V: Exploring meta-paths across multiple networks for lncRNA-disease association prediction,” in *Proceedings of the IEEE/ACM Transactions on Computational Biology and Bioinformatics*, Piscataway, NJ. 10.1109/TCBB.2019.2946257 31725386

[B15] Deng L. Wang J. Zhang J. (2019b). Predicting gene ontology function of human micrornas by integrating multiple networks. *Front. Genet.* 10:3 10.3389/fmicb.2018.0003 PMC6361788 30761178

[B16] Ding Y. Tang J. Guo F. (2017). Identification of drug-target interactions via multiple information integration. *Inform. Sci.* 418–419 546–560. 10.1016/j.ins.2017.08.045

[B17] Ding Y. Tang J. Guo F. (2019). Identification of drug-side effect association via multiple information integration with centered kernel alignment. *Neurocomputing* 325 211–224. 10.1016/j.neucom.2018.10.028

[B18] Dubchak I. Muchnik I. Holbrook S. R. Kim S. H. (1995). Prediction of protein folding class using global description of amino acid sequence. *Proc. Natl. Acad. Sci. U.S.A.* 92 8700–8704. 10.1073/pnas.92.19.8700 7568000 PMC41034

[B19] Fang T. Zhang Z. Sun R. Zhu L. He J. Huang B. (2019). RNAm5CPred: prediction of RNA 5-methylcytosine sites based on three different kinds of nucleotide composition. *Mol. Ther. Nucleic Acids* 18 739–747. 10.1016/j.omtn.2019.10.008 31726390 PMC6859278

[B20] Feng Y. M. (2019). Gene therapy on the road. *Curr. Gene Ther.* 19:6. 10.2174/1566523219999190426144513 31190640

[B21] Gong Y. Niu Y. Zhang W. Li X. (2019). A network embedding-based multiple information integration method for the MiRNA-disease association prediction. *BMC Bioinform.* 20:468. 10.1186/s12859-019-3063-3 31510919 PMC6740005

[B22] Guo M. Xu Y. (2018). Single-cell transcriptome analysis using SINCERA pipeline *Transcriptome*. *Data Analy.* 1751 209–222. 10.1007/978-1-4939-7710-9_15 PMC6699487 29508300

[B23] Hajisharifi Z. Piryaiee M. Mohammad Beigi M. Behbahani M. Mohabatkar H. (2014). Predicting anticancer peptides with Chou’s pseudo amino acid composition and investigating their mutagenicity via Ames test. *J. Theor. Biol.* 341 34–40. 10.1016/j.jtbi.2013.08.037 24035842

[B24] Hong J. Luo Y. Zhang Y. Ying J. Xue W. Xie T. (2019). Protein functional annotation of simultaneously improved stability, accuracy and false discovery rate achieved by a sequence-based deep learning. *Brief Bioinform.* 21 1437–1447. 10.1093/bib/bbz081 31504150 PMC7412958

[B25] Hong Z. Zeng X. Wei L. Liu X. J. B. (2019). Identifying enhancer-promoter interactions with neural network based on pre-trained DNA vectors and attention mechanism. *Bioinformatics* 36 1037–1043. 10.1093/bioinformatics/btz694 31588505

[B26] Huang Q. Chen Y. Liu L. Tao D. Li X. (2020). On combining biclustering mining and adaboost for breast tumor classification. *IEEE Trans. Knowl. Data Eng.* 32 728–738. 10.1109/TKDE.2019.2891622

[B27] Jia C. Z. Zuo Y. Zou Q. (2018). O-GlcNAcPRED-II: an integrated classification algorithm for identifying O-GlcNAcylation sites based on fuzzy undersampling and a K-means PCA oversampling technique. *Bioinformatics* 34 2029–2036. 10.1093/bioinformatics/bty039 29420699

[B28] Jiang Q. H. Wang G. H. Jin S. L. Li Y. Wang Y. D. (2013). Predicting human microRNA-disease associations based on support vector machine. *Intern. J. Data Min. Bioinform.* 8 282–293. 10.1504/ijdmb.2013.056078 24417022

[B29] Li B. Tang J. Yang Q. Li S. Cui X. Li Y. (2017). NOREVA: normalization and evaluation of MS-based metabolomics data. *Nucleic Acids Res.* 45 W162–W170. 10.1093/nar/gkx449 28525573 PMC5570188

[B30] Li F. Zhou Y. Zhang X. Tang J. Yang Q. Zhang Y. (2020). SSizer: determining the sample sufficiency for comparative biological study. *J. Mol. Biol.* 432:3411. 10.1016/j.jmb.2020.01.027 32044343

[B31] Li Y. H. Li X. X. Hong J. J. Wang Y. X. Fu J. B. Yang H. (2020). Clinical trials, progression-speed differentiating features and swiftness rule of the innovative targets of first-in-class drugs. *Brief Bioinform.* 21 649–662. 10.1093/bib/bby130 30689717 PMC7299286

[B32] Li W. Yu J. Lian B. Sun H. Li J. Zhang M. (2015). Identifying prognostic features by bottom-up approach and correlating to drug repositioning. *PLoS One* 10:e0118672. 10.1371/journal.pone.0118672 25738841 PMC4349868

[B33] Liang C. Changlu Q. He Z. Tongze F. Xue Z. (2019). gutMDisorder: a comprehensive database for dysbiosis of the gut microbiota in disorders and interventions. *Nucleic Acids Res.* 48 D554–D560. 10.1093/nar/gkz843 PMC6943049 31584099

[B34] Liao Y.-D. Jiang Z.-R. (2019). MoABank: an integrated database for drug mode of action knowledge. *Curr. Bioinform.* 14 446–449. 10.2174/1574893614666190416151344

[B35] Liao Z. J. Li D. P. Wang X. R. Li L. S. Zou Q. (2018). Cancer diagnosis through isomir expression with machine learning method. *Curr. Bioinform.* 13 57–63. 10.2174/1574893611666160609081155

[B36] Lin C. Chen W. Qiu C. Wu Y. Krishnan S. Zou Q. (2014). LibD3C: ensemble classifiers with a clustering and dynamic selection strategy. *Neurocomputing* 123 424–435. 10.1016/j.neucom.2013.08.004

[B37] Lin H. Liang Z. Y. Tang H. Chen W. (2017). Identifying sigma70 promoters with novel pseudo nucleotide composition. *IEEE/ACM Trans. Comput. Biol. Bioinform.* 16 1316–1321. 10.1109/TCBB.2017.2666141 28186907

[B38] Lin M. Li X. Guo H. Ji F. Ye L. Ma X. (2019). Identification of bone metastasis-associated genes of gastric cancer by genome-wide transcriptional profiling. *Curr. Bioinform.* 14 62–69. 10.2174/1574893612666171121154017

[B39] Liu B. Chen S. Yan K. Weng F. (2019a). iRO-PsekGCC: identify DNA replication origins based on pseudo k-tuple GC composition. *Front. Genet.* 10:842 10.3389/fmicb.2018.0842 PMC6759546 31620165

[B40] Liu B. Gao X. Zhang H. (2019b). BioSeq-Analysis2.0: an updated platform for analyzing DNA, RNA, and protein sequences at sequence level and residue level based on machine learning approaches. *Nucleic Acids Res.* 47:e127. 10.1093/nar/gkz740 PMC6847461 31504851

[B41] Liu B. Li K. (2019). iPromoter-2L2.0: identifying promoters and their types by combining smoothing cutting window algorithm and sequence-based features. *Mol. Ther.Nucleic Acids* 18 80–87. 31536883 10.1016/j.omtn.2019.08.008 PMC6796744

[B42] Liu H. Luo L. B. Cheng Z. Z. Sun J. J. Guan J. H. Zheng J. (2018). Group-sparse modeling drug-kinase networks for predicting combinatorial drug sensitivity in cancer cells. *Curr. Bioinform.* 13 437–443. 10.2174/1574893613666180118104250

[B43] Liu S. Liu C. Deng L. (2018). Machine learning approaches for protein-protein interaction hot spot prediction: progress and comparative assessment. *Molecules* 23:2535. 10.3390/molecules23102535 PMC6222875 30287797

[B44] Liu H. Zhang W. Zou B. Wang J. Deng Y. Deng L. (2020). DrugCombDB: a comprehensive database of drug combinations toward the discovery of combinatorial therapy. *Nucleic Acids Res.* 48 D871–D881. 31665429 10.1093/nar/gkz1007 PMC7145671

[B45] Liu X. Hong Z. Liu J. Lin Y. Rodríguez-Patón A. Zou Q. (2020). Computational methods for identifying the critical nodes in biological networks. *Briefings Bioinform.* 21 486–497. 10.1093/bib/bbz011 30753282

[B46] Liu W. Meng X. Xu Q. Flower D. R. Li T. (2006). Quantitative prediction of mouse class I MHC peptide binding affinity using support vector machine regression (SVR) models. *BMC Bioinform.* 7:182. 10.1186/1471-2105-7-182 16579851 PMC1513606

[B47] Lv H. Dao F.-Y. Zhang D. Guan Z.-X. Yang H. Su W. (2020). iDNA-MS: an integrated computational tool for detecting DNA modification sites in multiple genomes. *iScience* 23:100991. 10.1016/j.isci.2020.100991 32240948 PMC7115099

[B48] Lv H. Zhang Z. M. Li S. H. Tan J. X. Chen W. Lin H. (2019). Evaluation of different computational methods on 5-methylcytosine sites identification. *Briefings Bioinform.* 21 982–995. 10.1093/bib/bbz048 31157855

[B49] Lv Z. Jin S. Ding H. Zou Q. (2019). A random forest sub-Golgi protein classifier optimized via dipeptide and amino acid composition features. *Front. Bioeng. Biotechnol.* 7:215. 10.3389/fmicb.2018.00215 31552241 PMC6737778

[B50] Munir A. Malik S. I. Malik K. A. (2019). Proteome mining for the identification of putative drug targets for human pathogen clostridium tetani. *Curr. Bioinform.* 14 532–540. 10.2174/1574893613666181114095736

[B51] Ozkan A. Isgor S. B. Sengul G. Isgor Y. G. (2019). Benchmarking classification models for cell viability on novel cancer image datasets. *Curr. Bioinform.* 14 108–114. 10.2174/1574893614666181120093740

[B52] Qiao Y. Xiong Y. Gao H. Zhu X. Chen P. (2018). Protein-protein interface hot spots prediction based on a hybrid feature selection strategy. *BMC Bioinform.* 19:14. 10.1186/s12859-018-2009-5 29334889 PMC5769548

[B53] Qu K. Han K. Wu S. Wang G. Wei L. (2017). Identification of DNA-binding proteins using mixed feature representation methods. *Molecules* 22:1602. 10.3390/molecules22101602 28937647 PMC6151557

[B54] Ru X. Wang L. Li L. Ding H. Ye X. Zou Q. (2020). Exploration of the correlation between GPCRs and drugs based on a learning to rank algorithm. *Comput. Biol. Med.* 119:103660. 10.1016/j.compbiomed.2020.103660 32090901

[B55] Ru X. Q. Li L. H. Zou Q. (2019). Incorporating Distance-based top-n-gram and random forest to identify electron transport proteins. *J. Proteome Res.* 18 2931–2939. 10.1021/acs.jproteome.9b00250 31136183

[B56] Shan X. Wang X. Li C. D. Chu Y. Zhang Y. Xiong Y. (2019). Prediction of CYP450 enzyme-substrate selectivity based on the network-based label space division method. *J. Chem. Inf. Model.* 59 4577–4586. 10.1021/acs.jcim.9b00749 31603319

[B57] Shen J. Zhang J. Luo X. Zhu W. Yu K. Chen K. (2007). Predicting protein-protein interactions based only on sequences information. *Proc. Natl. Acad. Sci. U.S.A.* 104 4337–4341. 10.1073/pnas.0607879104 17360525 PMC1838603

[B58] Song B. Li K. Orellana-Martín D. Valencia-Cabrera L. Pérez-Jiménez M. J. (2020). Cell-like P systems with evolutional symport/antiport rules and membrane creation. *Inform. Comput.* 2020:104542.

[B59] Song T. Rodríguez-Patón A. Zheng P. Zeng X. (2018). Spiking neural P systems with colored spikes. *IEEE Trans. Cogn. Dev. Syst.* 10 1106–1115. 10.1109/TNB.2018.2873221 30281471

[B60] Srivastava N. Mishra B. N. Srivastava P. (2019). In-silico identification of drug lead molecule against pesticide exposed-neurodevelopmental disorders through network-based computational model approach. *Curr. Bioinform.* 14 460–467. 10.2174/1574893613666181112130346

[B61] Sun Y. Zhang W. Chen Y. Ma Q. Wei J. Liu Q. (2016). Identifying anti-cancer drug response related genes using an integrative analysis of transcriptomic and genomic variations with cell line-based drug perturbations. *Oncotarget* 7:9404. 10.18632/oncotarget.7012 PMC4891048 26824188

[B62] Sun Z. Deng Z.-H. Nie J.-Y. Tang J. (2019). Rotate: knowledge graph embedding by relational rotation in complex space. *arXiv* [Preprint]. arXiv:1902.10197v1

[B63] Tan J. X. Li S. H. Zhang Z. M. Chen C. X. Chen W. Tang H. (2019). Identification of hormone binding proteins based on machine learning methods. *Math. Biosci. Eng.* 16 2466–2480. 10.3934/mbe.2019123 31137222

[B64] Tang J. Fu J. Wang Y. Li B. Li Y. Yang Q. (2020). ANPELA: analysis and performance assessment of the label-free quantification workflow for metaproteomic studies. *Brief Bioinform.* 21 621–636. 10.1093/bib/bby127 30649171 PMC7299298

[B65] Tang J. Fu J. Wang Y. Luo Y. Yang Q. Li B. (2019). Simultaneous improvement in the precision, accuracy, and robustness of label-free proteome quantification by optimizing data manipulation chains. *Mol. Cell Proteom.* 18 1683–1699. 10.1074/mcp.RA118.001169 31097671 PMC6682996

[B66] Tang Y. Liu D. Wang Z. Wen T. Deng L. (2017). A boosting approach for prediction of protein-RNA binding residues. *BMC Bioinform.* 18:465 10.1186/s12859-018-2009-465 PMC5773889 29219069

[B67] Tyagi A. Kapoor P. Kumar R. Chaudhary K. Gautam A. Raghava G. P. (2013). In silico models for designing and discovering novel anticancer peptides. *Sci. Rep.* 3:2984. 10.1038/srep02984 24136089 PMC6505669

[B68] Wang H. Ding Y. Tang J. Guo F. (2020). Identification of membrane protein types via multivariate information fusion with Hilbert-Schmidt Independence criterion. *Neurocomputing* 383 257–269. 10.1016/j.neucom.2019.11.103

[B69] Wang J. Wang H. Wang X. Chang H. (2020). Predicting drug-target interactions via FM-DNN learning. *Curr. Bioinform.* 15 68–76. 10.2174/1574893614666190227160538

[B70] Wang Y. Zhang S. Li F. Zhou Y. Zhang Y. Wang Z. (2020). Therapeutic target database 2020: enriched resource for facilitating research and early development of targeted therapeutics. *Nucleic Acids Res.* 48 D1031–D1041. 10.1093/nar/gkz981 31691823 PMC7145558

[B71] Wang X. Yu B. Ma A. Chen C. Liu B. Ma Q. (2018). Protein-protein interaction sites prediction by ensemble random forests with synthetic minority oversampling technique. *Bioinformatics* 35 2395–2402. 10.1093/bioinformatics/bty995 PMC6612859 30520961

[B72] Wang Y. Ding Y. Guo F. Wei L. Tang J. (2017). Improved detection of DNA-binding proteins via compression technology on PSSM information. *PLoS One* 12:185587. 10.1371/journal.pone.0185587 28961273 PMC5621689

[B73] Wang Y. Liu K. Ma Q. Tan Y. Du W. Lv Y. (2019). Pancreatic cancer biomarker detection by two support vector strategies for recursive feature elimination. *Biomark. Med.* 13 105–121. 10.2217/bmm-2018-0273 30767554 PMC6737501

[B74] Wei L. Wan S. Guo J. Wong K. K. (2017). A novel hierarchical selective ensemble classifier with bioinformatics application. *Artif. Intellig. Med.* 83 82–90. 10.1016/j.artmed.2017.02.005 28245947

[B75] Wei L. Zhou C. Chen H. Song J. Su R. (2018). ACPred-FL: a sequence-based predictor based on effective feature representation to improve the prediction of anti-cancer peptides. *Bioinformatics* 34 4007–4016. 29868903 10.1093/bioinformatics/bty451 PMC6247924

[B76] Wei L. Zhou C. Su R. Zou Q. (2019). PEPred-Suite: improved and robust prediction of therapeutic peptides using adaptive feature representation learning. *Bioinformatics* 35 4272–4280. 10.1093/bioinformatics/btz246 30994882

[B77] Wei L. Zou Q. Liao M. Lu H. Zhao Y. (2016). A novel machine learning method for cytokine-receptor interaction prediction. *Combinat. Chem. High Throughput Screen.* 19 144–152. 10.2174/1386207319666151110122621 26552440

[B78] Wilcox R. (1961). Adaptive control processes—A guided tour, by richard bellman, princeton university press, princeton, New Jersey, 1961, 255 pp., $6.50. *Naval Res. Logist. Q.* 8:314 10.1002/nav.3800080314

[B79] Xiong Y. Wang Q. Yang J. Zhu X. Wei D. Q. (2018). PredT4SE-Stack: prediction of bacterial Type IV secreted effectors from protein sequences using a stacked ensemble method. *Front. Microbiol.* 9:2571. 10.3389/fmicb.2018.02571 30416498 PMC6212463

[B80] Xu L. Liang G. Liao C. Chen G.-D. Chang C.-C. (2018a). An efficient classifier for alzheimer’s disease genes identification. *Molecules* 23:3140. 10.3390/molecules23123140 PMC6321377 30501121

[B81] Xu L. Liang G. Wang L. Liao C. (2018b). A novel hybrid sequence-based model for identifying anticancer peptides. *Genes* 9:158. 10.3390/genes9030158 29534013 PMC5867879

[B82] Xu Y. Zhao W. Olson S. D. Prabhakara K. S. Zhou X. (2018). Alternative splicing links histone modifications to stem cell fate decision. *Genome Biol.* 19 1–21. 30217220 10.1186/s13059-018-1512-3 PMC6138936

[B83] Xu L. Liang G. Liao C. Chen G.-D. Chang C.-C. (2019). k-Skip-n-Gram-RF: a random forest based method for Alzheimer’s disease protein identification. *Front. Genet.* 10:33. 10.3389/fgene.2019.00033 30809242 PMC6379451

[B84] Xu Y. Wang Y. Luo J. Zhao W. Zhou X. (2017). Deep learning of the splicing (epi)genetic code reveals a novel candidate mechanism linking histone modifications to ESC fate decision. *Nucleic Acids Res.* 45 12100–12112. 10.1093/nar/gkx870 29036709 PMC5716079

[B85] Yan K. Fang X. Xu Y. Liu B. (2019). Protein fold recognition based on multi-view modeling. *Bioinformatics* 35 2982–2990. 30668845 10.1093/bioinformatics/btz040

[B86] Yang Q. Li B. Tang J. Cui X. Wang Y. Li X. (2019). Consistent gene signature of schizophrenia identified by a novel feature selection strategy from comprehensive sets of transcriptomic data. *Brief Bioinform.* 21 1058–1068. 10.1093/bib/bbz049 31157371

[B87] Yang Q. Wang Y. Zhang Y. Li F. Xia W. Zhou Y. (2020). NOREVA: enhanced normalization and evaluation of time-course and multi-class metabolomic data. *Nucleic Acids Res.* 48 W436–W448. 10.1093/nar/gkaa258 32324219 PMC7319444

[B88] Yin J. Sun W. Li F. Hong J. Li X. Zhou Y. (2020). VARIDT 1.0: variability of drug transporter database. *Nucleic Acids Res* 48 D1042–D1050. 10.1093/nar/gkz779 31495872 PMC6943059

[B89] Yu L. Gao L. (2019). Human pathway-based disease network. *IEEE/ACM Trans. Comput. Biol. Bioinform.* 16 1240–1249. 10.1109/TCBB.2017.2774802 29990107

[B90] Yu L. Xu F. Gao L. (2020). Predict new therapeutic drugs for hepatocellular carcinoma based on gene mutation and expression. *Front. Bioeng. Biotechnol.* 8:8. 10.3389/fbioe.2020.00008 32047745 PMC6997129

[B91] Zeng W. Wang F. Ma Y. Liang X. C. Chen P. (2019). Dysfunctional mechanism of liver cancer mediated by transcription factor and non-coding RNA. *Curr. Bioinform.* 14 100–107. 10.2174/1574893614666181119121916

[B92] Zeng X. Wang W. Deng G. Bing J. Zou Q. (2019a). Prediction of potential disease-associated MicroRNAs by using neural networks. *Mol. Ther. Nucleic Acids* 16 566–575. 31077936 10.1016/j.omtn.2019.04.010 PMC6510966

[B93] Zeng X. Zhu S. Liu X. Zhou Y. Nussinov R. Cheng F. (2019b). deepDR: a network-based deep learning approach to in silico drug repositioning. *Bioinformatics* 35 5191–5198. 10.1093/bioinformatics/btz418 31116390 PMC6954645

[B94] Zeng X. Liao Y. Liu Y. Zou Q. (2017). Prediction and validation of disease genes using hetesim scores. *IEEE/ACM Trans. Comput. Biol. Bioinform.* 14 687–695. 10.1109/tcbb.2016.2520947 26890920

[B95] Zeng X. Zhu S. Hou Y. Zhang P. Li L. Li J. (2020a). Network-based prediction of drug-target interactions using an arbitrary-order proximity embedded deep forest. *Bioinformatics* 36 2805–2812. 10.1093/bioinformatics/btaa010 31971579 PMC7203727

[B96] Zeng X. Zhu S. Lu W. Liu Z. Huang J. Zhou Y. (2020b). Target identification among known drugs by deep learning from heterogeneous networks. *Chem. Sci.* 11 1775–1797. 10.1039/C9SC04336E PMC8150105 34123272

[B97] Zhang J. Ju Y. Lu H. Xuan P. Zou Q. (2016). Accurate identification of cancerlectins through hybrid machine learning technology. *Int. J. Genom.* 2016:7604641. 10.1155/2016/7604641 27478823 PMC4961832

[B98] Zhang M. Li F. Marquez-Lago T. T. Leier A. Fan C. Kwoh C. K. (2019). MULTiPly: a novel multi-layer predictor for discovering general and specific types of promoters. *Bioinformatics* 35 2957–2965. 10.1093/bioinformatics/btz016 30649179 PMC6736106

[B99] Zhang W. Jing K. Huang F. Chen Y. Li B. Li J. (2019a). SFLLN: a sparse feature learning ensemble method with linear neighborhood regularization for predicting drug-drug interactions. *Inform. Sci.* 497 189–201. 10.1016/j.ins.2019.05.017

[B100] Zhang W. Li Z. Guo W. Yang W. Huang F. (2019b). “A fast linear neighborhood similarity-based network link inference method to predict microRNA-disease associations,” in *Proceedings of the IEEE/ACM Trans Comput Biol Bioinform*, Piscataway, NJ. 10.1109/TCBB.2019.2931546 31369383

[B101] Zhang Y. Kou C. Wang S. Zhang Y. (2019). Genome-wide differential-based analysis of the relationship between DNA methylation and gene expression in cancer. *Curr. Bioinform.* 14 783–792. 10.2174/1574893614666190424160046

[B102] Zhang W. Chen Y. Li D. Yue X. (2018a). Manifold regularized matrix factorization for drug-drug interaction prediction. *J. Biomed. Inform.* 88 90–97. 30445219 10.1016/j.jbi.2018.11.005

[B103] Zhang W. Yue X. Tang G. Wu W. Huang F. Zhang X. (2018b). SFPEL-LPI: sequence-based feature projection ensemble learning for predicting LncRNA-protein interactions. *PLoS Comput. Biol.* 14:e1006616. 10.1371/journal.pcbi.1006616 30533006 PMC6331124

[B104] Zhang Z. M. Tan J. X. Wang F. Dao F. Y. Zhang Z. Y. Lin H. (2020). Early diagnosis of hepatocellular carcinoma using machine learning method. *Front. Bioeng. Biotechnol.* 8:254. 10.3389/fbioe.2020.00254 32292778 PMC7122481

[B105] Zhou L. Y. Qin Z. Zhu Y. H. He Z. Y. Xu T. (2019). Current RNA-based therapeutics in clinical trials. *Curr. Gene Ther.* 19 172–196. 10.2174/1566523219666190719100526 31566126

[B106] Zou Q. Chen L. Huang T. Zhang Z. Xu Y. (2017). Machine learning and graph analytics in computational biomedicine. *Artif. Intell. Med*. 83:1. 10.1016/j.artmed.2017.09.003 28935226

[B107] Zou Q. Wan S. Ju Y. Tang J. Zeng X. (2016). Pretata: predicting TATA binding proteins with novel features and dimensionality reduction strategy. *BMC Syst. Biol.* 10:114 10.1186/s12859-018-2009-114 PMC5259984 28155714

