# Supplementary material for: Prediction of Anticancer Peptides Using a Low-Dimensional Feature Model
Source: Front Bioeng Biotechnol. 2020 Aug 12;8:892. doi: 10.3389/fbioe.2020.00892 (PMC7434836; doi:10.3389/fbioe.2020.00892)
Supplement: Supplementary file 1 [file Data_Sheet_1.PDF]

Supporting Information S1. The benchmark dataset contains 138 anticancer peptides and 206 non-anticancer peptides (see Eq.1).

I. 138 anticancer peptides

>ACP_1
GLWSKIKEVGKEAAKAAAKAAGKAALGAVSEAV
>ACP_2
GLFDIIKKIAESI
>ACP_3
GLLDIVKKVVGAFGSL
>ACP_4
GLFDIVKKVVGALGSL
>ACP_5
GLFDIVKKVVGTLAGL
>ACP_6
GLFDIAKKVIGVIGSL
>ACP_7
GLFDIVKKIAGHIAGSI
>ACP_8
GLFDIVKKIAGHIVSSI
>ACP_9
AACARFIDDFCDTLTPNIYRPRDNGQRCYAVNGHRCDFTVFNTNNGGNPIRASTPNCK
TVLRTAANRCPTGGRGKINPNAPFLFAIDPNDGDCSTNF
>ACP_10
HGVSGHGQHGVHG
>ACP_11
FKCRRWQWRMKKLGAPSITCVRRAF
>ACP_12
KWKLFKKIKFLHSAKKF
>ACP_13
KSSAYSLQMGATAIKQVKKLFKKWGW
>ACP_14
GIGTKILGGVKTALKGALKELASTYAN
>ACP_15
GIGGKILSGLKTALKGAAKELASTYLH
>ACP_16
GIGGVLLSAGKAALKGLAKVLAEKYAN
>ACP_17
SIGAKILGGVKTFFKGALKELASTYLQ
>ACP_18
FLPLLAGLAANFLPTIICKISYKC
>ACP_19
FVQWFSKFLGRIL

>ACP_20
KWKIFKKIEKVGRNIRNGIIKAGPAVAVLGEAKAL
>ACP_21
GWLKKIGKKIERVGQHTRDATIQTIGVAQQAANVAATLK
>ACP_22
KWKLFKKIEKVGQNIRDGIIKAGPAVAVVGQATQIAK
>ACP_23
SQLGDLGSGAGQGGGGGGSIRAAGGAFGKLEAAREEEFFYKKQKEQLERLKNDQIHQA
EFHHQQIKEHEEAIQRHKDFLNNLHK
>ACP_24
GIGKFLHSAKKFGKAFVGEIMNS
>ACP_25
GIGAVLKVLTTGLPALISWIKRKRQQ
>ACP_26
ALWKNMLKGIGKLAGQAALGAVKTLVGAE
>ACP_27
ACYCRIPACIAGERRYGTCIYQGRLWAFCC
>ACP_28
ECRRLCYKQRCVTYCRGR
>ACP_29
LKLKSIVSWAKKVL
>ACP_30
KWCFRVCYRGICYRRCR
>ACP_31
KSCCRNTWARNCYNVCRLPGTISREICAKKCDCKIISGTTCPSDYPK
>ACP_32
GLLSVLGSVAKHVLPHVVPVIAEHL
>ACP_33
GLLSVLGSVVKHVIPHVVPVIAEHL
>ACP_34
GLFKVLGSVAKHLLPHVAPVIAEK
>ACP_35
GLFGVLGSIAKHVLPHVVPVIAEK
>ACP_36
GLFVGVLAKVAAHVVPAIAEHF
>ACP_37
GLFVGLAKVAAHNNPAIAEHFQA
>ACP_38
GFVDFLKKVAGTIANVVT
>ACP_39
GLLQTIKEKLESLESLAKGIVSGIQA
>ACP_40
TRSSRAGLQFPVGRVHRLLRK
>ACP_41

FFGWLIKGAIHAGKAIHGLIHRRRH
>ACP_42
GLFDVIKKVASVIGGL
>ACP_43
GLFDIIKKVASVVGGL
>ACP_44
GRFKRFRKKFKKLFKKLSPVIPLLHLG
>ACP_45
GGLRSLGRKILRAWKKYGPIIVPIIRIG
>ACP_46
RRRPRPPYLPRPRPPPFFPPRLPPRIPPGFPPRFPPRFP
>ACP_47
GLLGPLLKIAAKVGSNLL
>ACP_48
GLICESCRKIIQKLEDMVGPQPNEDTVTQAASQVCDKLKILRGLCKKIMRSFLRRISWDI
LTGKKPQAICVDIKICKEKTGLI
>ACP_49
DHYNCVSSGGQCLYSACPIFTKIQGTCYRGKAKCCK
>ACP_50
FFHHIFRGIVHVGKTIHRLVTG
>ACP_51
KLAKLAKKLAKLAK
>ACP_52
KTCENLADTFRGPCFATSNC
>ACP_53
IDWKKLLDAAKQIL
>ACP_54
FLIGMTQGLICLITRKC
>ACP_55
ILPILSLIGGLLGK
>ACP_56
GLLGLLGSVVSHVVPAIVGHF
>ACP_57
GLLGLLGSVVSHVLPAITQHL
>ACP_58
GIKCRFCCGCCTPGICGVCCRF
>ACP_59
QSHLSLCRWCCNCCRSNKGC
>ACP_60
ILGPVISTIGGVLGGLLKNL
>ACP_61
FLPILASLAAKFGPKLFCLVTKKC
>ACP_62
GLWSKIKEAAKAAGKAALNAVTGLVNQGDQPS

>ACP_63
LLGMIPLAISAISALSKL
>ACP_64
GLPVCGETCVGGTCNTPGCSCSWPVCTRN
>ACP_65
GVPICGETCTLGTCYTAGCSCSWPVCTRN
>ACP_66
GIPCGESCVWIPCISSAIGCSCKSKVCYRN
>ACP_67
GIPCAESCVWIPCTVTALIGCGCSNKVCYN
>ACP_68
GTFPCGESCVFIPCLTSAIGCSCKSKVCYKN
>ACP_69
GLLPCAESCVYIPCLTTVIGCSCKSKVCYKN
>ACP_70
GRDYRTCLTIVQKLKKMVDKPTQRSVSNAATRVCRTGRSRWRDVCRNFMRRYQSRVT
QGLVAGETAQQICEDLRLCIPSTGPL
>ACP_71
GETDPNTQLLNDLGNNMAWGAALGAPGGLGSAALGAAGGALQTVGQGLIDHGPVNV
FIPVLIGPSWNGSGSGYNSATSSSGSGS
>ACP_72
GFKDLLKGAAKALVKTVLF
>ACP_73
KSCCPNTTGRNIYNACRLTGAPRPTCAKLSGCKIISGSTCPSDYPK
>ACP_74
KSCCPNTTGRNIYNTCRFGGGSREVCARISGCKIISASTCPSDYPK
>ACP_75
KSCCPNTTGRNIYNTCRLTGSSRETCAKLSGCKIISASTCPSNYPK
>ACP_76
MRKEFHNVLSSGQLLADKRPARDYNRK
>ACP_77
KSCCKNTTGRNIYNTCRFAGGSRERCAKLSGCKIISASTCPSDYPK
>ACP_78
FIFHIIKGLFHAGKMIHGLVTRRRH
>ACP_79
FLPAIVGAAAKFLPKIFCAISKKC
>ACP_80
FLPIIAGVAAKVLPKIFCAISKKC
>ACP_81
FLPIIAGIAAKFLPKIFCTISKKC
>ACP_82
FLPVIAGVAANFLPKLFCAISKKC
>ACP_83
FLPIIAGAAAKVVQKIFCAISKKC

>ACP_84
GLMDTIKGVAKTVAASWLDKLKCKITGC
>ACP_85
VNWKKVLGKIIKVAK
>ACP_86
VNWKKILGKIIKVAK
>ACP_87
FFSLLPSLIGGLVSAIK
>ACP_88
RFRLPFRRPPIRIHPPPFYPPFRRFL
>ACP_89
KWKLFKKIPKFLHLAKKF
>ACP_90
YKQCHKKGGHCFPKEKICLPPSSDFGKMDCRWRWKCCKKGSG
>ACP_91
GIPCGESCVFIPCITGAIGCSCKSKVCYRN
>ACP_92
GEFLKCGESCVQGECYTPGCSCDWPICKKN
>ACP_93
GLPTCGETCTLGTCYVPDCSCSWPICMKN
>ACP_94
GLPVCGETCFGGTCNTPGCTCDPWPVCTRN
>ACP_95
FVDLKKIANIINSIFGK
>ACP_96
GSIPCGESCVFIPCISSVIGCACKSKVCYKN
>ACP_97
GIPCGESCVFIPCISSVIGCSCSSKVCYRN
>ACP_98
GSIPCGESCVFIPCISAVIGCSCSNKVCYKN
>ACP_99
GSIPCEGSCVFIPCISAIIGCSCSNKVCYKN
>ACP_100
GIPCGESCVFIPCLTSAIDCSCKSKVCYRN
>ACP_101
GMWSKILGHLIR
>ACP_102
GKWMSLLKHILK
>ACP_103
GFGMALKLLKKVL
>ACP_104
GTGLPMSERRKIMLMMR
>ACP_105
GIACGESCVFLGCFIPGCSCKSKVCYFN

>ACP_106
GVIPCGESCVFIPCISSVLGCSCKNKVCYRD
>ACP_107
KLCGETCFKFKCYTPGCSCSYPFCK
>ACP_108
GDACGETCFTGICFTAGCSCNPWPTCTRN
>ACP_109
GIPCAESCVWIPPCTITALMGCSCKNNVCYNN
>ACP_110
IPCGESCVWIPCITAIAGCSCKNKVCYT
>ACP_111
AIPCGESCVWIPCISTVIGCSCSNKVCYR
>ACP_112
GEYCGESCYLIPCFTPGCYCVSRQCVNKN
>ACP_113
IPCGESCVWIPCISGMFGCSCKDKVCYS
>ACP_114
FLGWLFKWASK
>ACP_115
FLKWLFKWAKK
>ACP_116
KWKSFLKTFKSAKKTVLHTALKAISS
>ACP_117
KWKSFLKTFKSLKKTVLHTLLKAISS
>ACP_118
MPFLFCNVNDVCNFASRNDYSCNYYSNSYSFWLASLNPER
>ACP_119
KWKLFKKIGAVLKVL
>ACP_120
GACFSIAHECGA
>ACP_121
TCCATGACGTTCCTGACGTT
>ACP_122
KRFKQDGGASHASPASS
>ACP_123
KRAKAAGGWSHWSPWSSC
>ACP_124
LLGDFFRKSKEKIGKEFKRIVQRIKDFLRNLVPRTES
>ACP_125
FLGALFKVASKVLPSVKCAITKKC
>ACP_126
GIGKFLKKAKKFGKAFVKILKK
>ACP_127
GIGKFLKKAKKGIGAVLKVLTTGL

>ACP_128
VECYGPNRPQF
>ACP_129
KRFKQDGGWSHWSPWSSC
>ACP_130
RQVFQVAYIIIKA
>ACP_131
KAFDITYVRLKF
>ACP_132
DFKLFAVTIKYR
>ACP_133
DFKLFAVYIKYR
>ACP_134
WHSDMEWWYLLG
>ACP_135
HTMYYHHYQHHL
>ACP_136
RLVSYNGIIFFLK
>ACP_137
GRENYHGCTTHWGFTLC
>ACP_138
ASSSYPLIHWRPWAR

II. 206 non-anticancer peptides
>non-ACP_1
MTISLIWGIAMVVCCCIWVIFDRRRRKAGEPPL
>non-ACP_2
MFATPLRQPTNASGARPAVSMDGQETPFQYEITD
>non-ACP_3
LLWRKVAGATVGPGPVPA
>non-ACP_4
DSPDPMNGASSNALIAKMNSAKLLYQHY
>non-ACP_5
NNQEVIDAISQAISQTPGCVL
>non-ACP_6
KKVVEKNADPETTLLVYLRRKLGLCGTKLGCGEG
>non-ACP_7
CSRLLPSLAQEEG
>non-ACP_8
KNDFAALQAKLDADAAEIEKWWSDSR
>non-ACP_9
VDREQLVQKARLAEQAERYDD
>non-ACP_10
RPLRRVVLFYQGKLCSMAGNFWQSSHYLQW

>non-ACP_11
GAAGERKLCLLSLLLIGA
>non-ACP_12
MFRKLLKMWILLRPTHWLILIALCAVTCAGYWLLWSE
>non-ACP_13
HLRGPADSGWMPQAAPCLSGAPQAS
>non-ACP_14
XSARLTVLLRHLGCRSAGTI
>non-ACP_15
NNPNNSNSHLRPHAYNNSRRDDSD
>non-ACP_16
VVILASLSVMFLVSLWQQKIRERLPPGPTPLPFIGNY
>non-ACP_17
ICLSCLISFFLWNQNRAKGKLPPG
>non-ACP_18
VVMNSLRVILQAS
>non-ACP_19
ARPRLDLQLVQRFVRIQKVF
>non-ACP_20
MLAKGLSLRSVLAKGCQPFLSPTWQSSVLATGGGANIS
>non-ACP_21
AAGLPGAALPLRKRPLRAPSPEPAAPRGAAGLVV
>non-ACP_22
PPMPSAPPVHPPP
>non-ACP_23
SCPIDKRRPLIAFLRRLRD
>non-ACP_24
RLGLWASGLILILGFLKLLRLLLRRQRLARAMD
>non-ACP_25
FSPQRDRFQAEGS
>non-ACP_26
GTLWALVFLGILVGMVVPSPAGTRANNTLLDSRG
>non-ACP_27
MNRLLQKGTSLVPSWRTR
>non-ACP_28
MTTSLIWGIAIAACCCLWLILGIRRRQT
>non-ACP_29
ATLANGMSLQPPLEEVS
>non-ACP_30
PLTATNSGLAVNN
>non-ACP_31
VRACHKVCRCLLSGFGGRVDAGQPELLTER
>non-ACP_32
TAGILLLLLLGTLEGS

>non-ACP_33
MEPSILLLLALLVGFLLLLVRGH
>non-ACP_34
MKNCFQLLCNLKVPAAGFKNTVKS
>non-ACP_35
SVIHTQAHASGLQQVPQLVPAGPGGGGKAVAPSKQSK
>non-ACP_36
KPLGLLKPSSLMKVSGRFKAHQDA
>non-ACP_37
ARTLNNKLSLSKPKFSGFT
>non-ACP_38
LLLVIIWTLFGPSGLGEELLSLSLASLLPAPASPGPP
>non-ACP_39
WPGILVGGARVASCRYPALGPRLA
>non-ACP_40
RSVKGLVALITGGASGL
>non-ACP_41
AAAALRARILQVSSKVN
>non-ACP_42
TGCCIAGRLANLDDQNLTVAL
>non-ACP_43
GSILGFLQIATVLTVLLLLLK
>non-ACP_44
AARQIGSCLMRCRTLDTTSP
>non-ACP_45
WGCRGRRWAFARVDGGSCHRRGAPTGSTSNQIR
>non-ACP_46
YAKPGAVRSPAQILQWQVLPNTVPAKS
>non-ACP_47
RMAGPWLSLHEARLLGTRGAAAPKAV
>non-ACP_48
SISNRAAVPEHGVAPDAERL
>non-ACP_49
PNFSMETWLLLV
>non-ACP_50
PRPPSKTYRGAFQN
>non-ACP_51
SVLVKGCQPFLSAPRECPGHPRVGT
>non-ACP_52
LVTPPKALLKPLSIPNQ
>non-ACP_53
KMQGSRMDEQRCS
>non-ACP_54
VIADDLPPTCIRP

>non-ACP_55
LPGGLRVLVQTGH
>non-ACP_56
GWIWRWGWGRRCLGRPGLPGPGPGPATPLFLLLL
>non-ACP_57
RGIRGSSAARPSGRRRDPAGRTTETGFNIFTQHD
>non-ACP_58
QQEKEFLESYPQNCPPDALPGTPGNLD
>non-ACP_59
APARRVLQVKRVMQESSLSPAHL
>non-ACP_60
KVAPGGPTGYPGNLTAEQEQKLGELKMILL
>non-ACP_61
FLASYPQKCPAGSLPGTPGNTDE
>non-ACP_62
MDAKARNCLLQHREALEKDIKTSY
>non-ACP_63
ASRQLLVAPPEAL
>non-ACP_64
MISNGIGTVTTGKRSMCLFPLLLIGLWGC
>non-ACP_65
MTLRNFGMGKRSIEDRVQEEARCLVEELRKTNASPC
>non-ACP_66
AVFGLGGVGLSVIMGCKAAGASRIIAVDIN
>non-ACP_67
PNAKQSILQKNPDDVVIVAAYRTA
>non-ACP_68
AMELLLTATIFYLVLWVVKAFRLQVPKGLKSPPGP
>non-ACP_69
LLAAGFCPAVLCH
>non-ACP_70
AASVNDEQHQRIIKYGRALVLDIVEQ
>non-ACP_71
IARLREDGIQKRVIQEGRGELPDFQDG
>non-ACP_72
FIVVMNILALTLPFLAAEVQN
>non-ACP_73
CQNGRRANRTVRFARTA
>non-ACP_74
WVTVRSQQRGLFPAI
>non-ACP_75
LLRSCPLQGSPGRPRSV
>non-ACP_76
LNDGHFMPVLGFGTYAPPEVPRNRAVEV

>non-ACP_77
HLGRPSAPTIVAQPVSGLASPASFQPEQFQYTLDNNVLT
>non-ACP_78
RPEPGGCCCRRTVRANGC
>non-ACP_79
SWVEENRASFQPPVCNKLMHR
>non-ACP_80
VFHRVRWAPELGASLG
>non-ACP_81
RAKVLTLDGMNPRVRRVEYAVRGPIVQRALELEQELRQ
>non-ACP_82
LLQRARLAEQAERYDDMASAMKAVTELNEPLS
>non-ACP_83
ALIQKLNSDPQFVLAQNVGTTHDLLDICLKRATVQRA
>non-ACP_84
AALPMLWTGLVLLGLLGFPQTPAQGHDTVQPNFQQ
>non-ACP_85
QRRQNDSSVFLAIMVAAAVES
>non-ACP_86
CNAPGCGQRFTNEDHLAVHKHKHEMTLKFGPARTDS
>non-ACP_87
LVLLTVQNSALILTLNYSRIMPGYD
>non-ACP_88
TVLSPPQRFKRILQAMMLAVAVV
>non-ACP_89
ISRGLLLLAALCCLAPSFL
>non-ACP_90
VAGTESAQGPPGPAASLELWLNKATDPS
>non-ACP_91
QYLRIRTVQPEPDYGAAV
>non-ACP_92
ASPTQTPPTTSTIRVARRSRVALVAM
>non-ACP_93
TIWRNQHTYKMATSASANLSKIVKKNYMELPQDGKVQ
>non-ACP_94
LSITRGLLLLAALCCLAPIS
>non-ACP_95
ILLSIWRQSSGRGKLPPGPIPLPIIGNIFQ
>non-ACP_96
LLLLSWVALGPRSLEGADPGTPGEAEGPACP
>non-ACP_97
LRVKRAMQEASFMPPLLPPAAHQRFSTVPAVP
>non-ACP_98
GLLLLAGLCCLVFGIMAEDAQVAQGPSQQI

>non-ACP_99
RHVGLLCATGPQRWRF
>non-ACP_100
AAVALARPKPPLRHQEHLQNEPDS
>non-ACP_101
SRVNDQSQASRNGLKGKVLTLDTMNPCV
>non-ACP_102
AAALGPGVLQATRAFHTGQPRLAPLPPLPEYGGK
>non-ACP_103
LCTSGLWTAQASTNESSNSHRGLAPTNV
>non-ACP_104
PAIQPVLSGLSRIVNGEEA
>non-ACP_105
GCQASLSTAQERLGHPGVPTREGVR
>non-ACP_106
RKVLILTLVVAACGFVLWSSNGR
>non-ACP_107
GRVRSRCPGPALLLLLALAARPALAGPPAAALQ
>non-ACP_108
CRITKPALLVLNQETAKVVQT
>non-ACP_109
KAEVCMAVPWLSLQ
>non-ACP_110
SHLELNNGTKMPTLGLGT
>non-ACP_111
LLLPEAAAERDAREKLALWDRRPDTTAPL
>non-ACP_112
LLALSLVLLYRYATYSHGFFKKLGIPGPKPLPLFGNVLS
>non-ACP_113
LLSLWRQSSGRGKLPPGPTPLPVIGNILQIGIKD
>non-ACP_114
AFKSMEVANFYYEADCLAAAYGGKAAPAAPPADRPGPR
>non-ACP_115
SLFWAARPLQRCGQLVRMAIRAQH
>non-ACP_116
MRPPRTLTSTATMSALSTSMPMEIDDVMDEDAVNGQA
>non-ACP_117
LLSLIGFCWAQYDP
>non-ACP_118
LWARSKNDQLRISFPPGLCWG
>non-ACP_119
PQGFDVDRDAKKLNKACKGMGTNEAAIIEILSG
>non-ACP_120
IFCLILWVVKAWQPRLPKGLKSPPGPWGWPLLG

>non-ACP_121
LDAASPGPLALLGLLFAATLLLSALFLL
>non-ACP_122
VTLLFKLYCLA
>non-ACP_123
ATRAAAARLVGTAASRTPAAARH
>non-ACP_124
RNKLDLETLTDILEHQIR
>non-ACP_125
RRLVLQARTYAQAAASPAPAAGPGQMSFTFASPTQVFF
>non-ACP_126
PGRSRSAADDINPAPANM
>non-ACP_127
LLSALTLETWVLLAVILVLLYRLG
>non-ACP_128
VMGHGLCPQGARAKAAIPAALRDHEST
>non-ACP_129
FLVSIAGLLYALVQLGQPCDCLPPLRAAA
>non-ACP_130
VRSVRAAVGGLRAISAPSAPCLPRPWGLRAG
>non-ACP_131
RGGCWPRGLQQLLVPGG
>non-ACP_132
APATPPRPLKRKKLQFTDVTPESSP
>non-ACP_133
EQAERYDDMAAAMKAVTEQGHELSNEERNL
>non-ACP_134
MPNDPSDNQLK
>non-ACP_135
TELLLAITVFCLGFWVVRALRTQVP
>non-ACP_136
LTADLLGAPFFTLPKELQLALLERQTVFL
>non-ACP_137
GHGRLVEIQGRLGVRIER
>non-ACP_138
LPFKLLLFVLLDGWTRLTH
>non-ACP_139
QALWLVLVLSMPPVLVAAVVGTLVSLVQ
>non-ACP_140
TASWLGNELHSFAEMTMMKIQGIR
>non-ACP_141
KHRTVLFRRWMAIICCLI
>non-ACP_142
LHRAQDYRRELDTLQSLLTTSQSSELQAAAALLKC

>non-ACP_143
TKGNDIADLDAVAQTLKKPADDANKAVN
>non-ACP_144
QLEMALAKLKKDMMRGGDAKQYQVWQS
>non-ACP_145
VSVDNPNDLMLMQWSLIRITIQEE
>non-ACP_146
QGKAGEVIVTGLRTEQLSLSDAKLLLEAAMR
>non-ACP_147
RGVGGPSQQVMTPQGRGTVAAAAAAATASIAGAP
>non-ACP_148
VGKSSKMLQHIDYRMRCILQDGRIFIGTFKA
>non-ACP_149
HHKDSSIHHLRLSANDAEDSLRM
>non-ACP_150
LNPDPCKPLAFI
>non-ACP_151
SLINQQITQVGHGGQAGRLTETNPLTENS
>non-ACP_152
IACLLPRVSNLELKQGKAGEVIVTGLR
>non-ACP_153
EPSTGLMLVEESRWVHLGRELLPATAP
>non-ACP_154
NATSSQDQFAKWARLNRKYEQLSQ
>non-ACP_155
EPQKTIWRLAIFLHLPLRLAVAKIY
>non-ACP_156
LFLVNVLAFGLAGYCFVRHNARCEAGV
>non-ACP_157
PTYERLNDPKNVFFRMPFARL
>non-ACP_158
WNHYLSCASPCPGYRLLCRIN
>non-ACP_159
EAAMAAAAAPGSGNGVGGGGGTAG
>non-ACP_160
LGSGHHDGSGDSLEMSSLDRAQ
>non-ACP_161
FYQNRAAAFEQLQKWKEVAQDCT
>non-ACP_162
ELNPKYVKALFRRAKAHE
>non-ACP_163
MAEALLLRATFYLLIGSANAAKPD
>non-ACP_164
RANALIKRGTMCMQQQQPMLSTQ

>non-ACP_165
RPKFALAQAQKCFALYRQAYTANNSSQVQAAMKGFEE
>non-ACP_166
YAQALTDQQQFGKADEMYDKCI
>non-ACP_167
GNMEKAIDMFNKAINLAKSEMEMAHLYSLC
>non-ACP_168
LSVALGPRSRSYRAPPPPRRRPGPHS
>non-ACP_169
MQESLRLQQQALEAR
>non-ACP_170
RKRLKEERQRQKKEARIAA
>non-ACP_171
GETRPSECIAALFDDRARPP
>non-ACP_172
LLEFAQLHNISSRTLQRQFVAS
>non-ACP_173
AASVNPLAAVELQRLSAQER
>non-ACP_174
QNCVFADNIKVGQMTEPLKDQQIIL
>non-ACP_175
LTMMAPAVAQQTDDETFV
>non-ACP_176
QQIQGGKELKDALAQL
>non-ACP_177
ERFTSNQMFFDLAQASASGGLNNKKI
>non-ACP_178
RQQEFLLLNGWLQLQCGHAERACILLD
>non-ACP_179
NWLCLSRAQQLNGDLDKARHAYQ
>non-ACP_180
YKELYYRLLTSPAADAGNTL
>non-ACP_181
EWRDLKGRADINKDDVV
>non-ACP_182
GWVTTLGPGSRGYRAPPPPRRSRE
>non-ACP_183
QMIENWRRQQQARREKAQADKERRARLQ
>non-ACP_184
QRKKKEARAAAMAAAAAQDPADSETP
>non-ACP_185
APATRSRYLLRLTVTLGPRSRSYHAPPPPRRRP
>non-ACP_186
ENWRKQKRERWEKIQADKERRARLQAEAQE

>non-ACP_187
LKEERQRQKKEARIAAMASAEGQDSAEAQD
>non-ACP_188
TLDRDGTLVRLRFTMVALITVCCPLVAFFF
>non-ACP_189
LFLTGCKVDLYTGISQKEGNEMLALL
>non-ACP_190
LDRDGTLVRLRFTLVALVTVCCPLVAFLFCVL
>non-ACP_191
LWRLCIGLHSAPRFLVAMAYLKYYQGTPC
>non-ACP_192
VAGTTAVAGQVPIMGAVKFGGDVCAAG
>non-ACP_193
AKLRRKHDKAMDEYEAMNKKLTAQK
>non-ACP_194
QLRSRSASPASTAKAQKAQ
>non-ACP_195
LIWTIFILHIAIFLVNTIGAATIDNLLWLLYLKLP
>non-ACP_196
PDPPADSPLDQTIQHLQGLTIQELPDPPTHLPE
>non-ACP_197
ERFAQLCEEHGILRENIIDLSNANRCL
>non-ACP_198
EEGLQGKLRLLRLLHQTNPYPQGPGTASQRRNRRRRRR
>non-ACP_199
PTDSPLDRAIQHLQRLTIQELPDPPTDLPESNSNQ
>non-ACP_200
GPGTANQRRQRRRRWRRRWQQLLALA
>non-ACP_201
EEELRKRLRLIHLLHQTIDSYPTGPGTANQRRQRR
>non-ACP_202
TDTPLDLAIQQLQNLAIESIPDPPTNTPEALCD
>non-ACP_203
LRLIHFLHQTTDPYPQGPGTANQRRRR
>non-ACP_204
PVDTPLDLAIQQLQGLAIEELPDPPTSAPEPLNDV
>non-ACP_205
NTNVTPHLLAGMRLIAVQQPEDPLRVL
>non-ACP_206
QTLVHNGGRLPPDLQLSAEDSSSTPS
